# The Relationship of Depth of Anaesthesia With Blink Reflex in Cats

**DOI:** 10.1002/vms3.70366

**Published:** 2025-04-22

**Authors:** Özlem Şengöz Şirin, Ayşe Nihan Elvan

**Affiliations:** ^1^ Department of Surgery Faculty of Veterinary Medicine, Burdur Mehmet Akif Ersoy University Burdur Turkey; ^2^ Institute of Health Sciences Burdur Mehmet Akif Ersoy University Burdur Turkey

**Keywords:** cat, electromyography, eye, minimum alveolar concentration, orbicularis oculi

## Abstract

**Background:**

The blink reflex is a neurological response characterised by eyelid movements and can provide important data on the state of anaesthesia. Sevoflurane is a commonly used inhalation anaesthetic agent in cats and evaluation of eye reflexes under this agent may help to better understand the depth of anaesthesia.

**Objectives:**

The aim of this study was to evaluate the relationship between the blink reflex and the depth of anaesthesia in cats and to evaluate the parameters that can be obtained from the blink reflex in addition to its effect in determining the depth of anaesthesia.

**Methods:**

All cats were premedicated with midazolam 0.1 mg kg^−1^ and butorphanol 0.4 mg kg^−1^. Propofol at a dose of 4 mg kg^−1^ was administered for the induction of anaesthesia. Following this, anaesthesia was maintained with sevoflurane. Anaesthesia was maintained at a minimum alveolar concentration (MAC) value of 2.9, which is the end‐tidal MAC value that provides deep anaesthesia for cats. Following the end of the operation, electrical stimulation was given to the supraorbital nerve for each 0.1 for each decreasing MAC value starting from the end‐tidal MAC value of 2.9, and blink parameters were recorded from the orbicularis oculi muscle. In this study, the mean latency, amplitude and duration values in sevoflurane anaesthesia in cats were determined.

**Results:**

In cats, only two blink responses, R1 and R2, were found in all MAC values. At the same time, a very strong positive correlation was found between R1 latency and MAC, and a moderate negative correlation was found between R2 latency and MAC. There was a very strong negative correlation between R1 amplitude and MAC and a moderate positive correlation between R2 amplitude and MAC. There was a moderate negative correlation between R1 duration and MAC and a weak negative correlation between R2 duration and MAC. When the relationship between anaesthesia stages and MAC was compared, R1 amplitude and R2 amplitude were found to be significant only between stage II and awake stage. In all other stages, R1 latency, R2 latency, R1 amplitude and R2 amplitude were not significant. All cats woke up with an average MAC value of 0.43 ± 0.20.

**Conclusions:**

In this study, mean latency, amplitude and duration values in cats under sevoflurane anaesthesia were determined. It was demonstrated that it was possible to elicit blink parameters in cats under sevoflurane anaesthesia with a short stimulus sequence to the supraorbital nerve. Only two blink responses, R1 and R2, were elicited at all MAC values.

## Graphical abstract

This study investigates the relationship between the blink reflex and anesthesia depth in cats under sevoflurane anesthesia. Using electrical stimulation of the supraorbital nerve, we identified two blink responses, R1 and R2, at all FeSEVO levels. Strong correlations were observed between FeSEVO and blink reflex parameters, including latency, amplitude, and duration. The findings suggest that monitoring the blink reflex could serve as an indicator of anesthesia depth in feline patients.

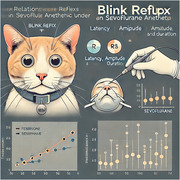

## 1 Introduction

Electromyography (EMG) is an electrodiagnostic method in which the electrical activity (potentials) of the patient's muscle fibres and nerves is detected and recorded. Blinking is an indispensable part of everyone's daily life (Smit 2008). Blinking is a movement that occurs when both the upper and lower eyelids temporarily close (Espinosa et al. 2018). About 100 years ago, the English doctor Overend revealed the blink reflex by tapping one side of the forehead. The mechanisms underlying this reflex remained unclear until Kugelberge (1952) analysed the blink reflex electromyographically by electrically stimulating the supraorbital nerve. He showed that the reflex consists of two responses. The first or early response, R1, is unilateral and occurs on the same side of the side on which the supraorbital nerve is stimulated. This response is not seen clinically. The second or late response, R2, is bilateral and occurs after a latent period. R2 responses cause actual closure of the eyelids (Aramideh et al. 1997). Depth of anaesthesia is the degree of suppression of the central nervous system (CNS) by a general anaesthetic, depending on the strength of the anaesthetic and the concentration at which it is administered. Arthur Ernest Guedel (1937) described a detailed classification of the anaesthetic state based on the use of a single inhalation anaesthetic agent, diethyl ether (Rani and Harsoor 2012). The aim of this master's thesis was to evaluate the relationship between the blink reflex and the depth of anaesthesia in cats and to evaluate the parameters that can be obtained from the blink reflex in addition to its effect in determining the depth of anaesthesia.

## 2 Materials and Methods

### 2.1 Ethical Approval

The animals used in this study were sourced from the Burdur Mehmet Akif Ersoy University. Burdur Mehmet Akif Ersoy University Local Board of Ethics Committee for Animal Experiments has approved the study protocol of this research (decision no: 2023/1178). The authors have followed ARRIVE guidelines to protect animals used for scientific purposes.

### 2.2 Animals

In this study, 7 females and 25 males, all of which were mongrel breeds, with normal cranial nerve examination, without any ophthalmological problem, in class I or II according to ASA (American Society of Anaesthesiologists) classification, were brought to XX as animal material and operated with various indications. The body weights of the cases were between 1.8 and 5 kg (mean 3.80 ± 0.8) and the ages were between 6 and 48 years (months) (mean 18.68 ± 11.82).

### 2.3 Materials Used in Anaesthesia

An intravenous access was established with an appropriately sized branula to anaesthetise the patient. Cefazolin (22 mg/kg, IV) was administered intravenously at a dose of 22 mg kg^−1^ 30 min before the patients were anaesthetised.

For premedication, midazolam (Zolamid 5 mg/5 mL) was administered intravenously at a dose of 0.1 mg kg^−1^ and butorphanol (Butorphanol hydrogen tartrate, 10 mg kg^−1^, Butomidor) was administered intravenously at a dose of 0.4 mg kg^−1^. Propofol (Propofol 1%, Polifarma, Turkey) was administered intravenously at a dose of 4 mg kg^−1^ for induction. Following induction, the patient was intubated with an endotracheal tube of the appropriate size and connected to the anaesthesia machine (Dräger, Primus, Lübeck, Germany). Sevoflurane (Sojourn 100% inhalation solution) was used to maintain anaesthesia by adjusting the end‐tidal minimal alveolar concentration (MAC) value so that the patient entered deep anaesthesia until the end of the operation. In addition, 0.9% isotonic sodium chloride ((Polifarma İlaç San. ve Tic. A.Ş., Tekirdag, Turkey) was infused at a dose of 3 mL kg^−1^ hour in all cases.

### 2.4 Materials Used in Perioperative Data Collection

In the cats included in the study, post‐intubation cardiovascular parameters (end‐tidal carbon dioxide, tidal volume, compliance [degree of lung expansion for each unit increase in pressure, Cpat], expiratory MAC, minute volume [MV]) were recorded using an inhalation anaesthesia device (Dräger, Primus Lübeck, Germany).

Respiratory rate, non‐invasive blood pressure (NIBP‐mmHg), systolic arterial pressure (SAP‐mmHg), diastolic arterial pressure (DAP‐mmHg), mean arterial pressure (MAP‐mmHg), body temperature and arterial oxygen saturation (SpO2‐pulse oximetry) values were monitored and recorded with a bedside monitor (URIT, UC50, Guilin, Guangxi, China) before and during anaesthesia.

Following the end of the operation, the needle electrodes were placed in the patients who were anaesthetised to a sufficient depth and the vaporiser was switched off. Starting from 2.9% end‐tidal MAC, at each decreasing MAC value of 0.1%, the right supraorbital nerves of the patients were electrically stimulated using an EMG device (Medelec Synergy, Oxford Instruments, Abingdon, UK) and blink reflex recordings were obtained. At the same time, the stages of anaesthesia were scaled according to Guedel classification, and the blink reflex parameters were recorded at which stage of anaesthesia.

### 2.5 Intraoperative Data Collection

Cardiovascular parameters (pulse rate, blood pressure, respiratory rate, end‐tidal carbon dioxide, partial oxygen pressure, tidal volume [respiratory volume], MAC, end‐tidal sevoflurane, MV) and arterial oxygen saturation values were measured at 5 min intervals after intubation and recorded on the anaesthesia follow‐up form.

### 2.6 EMG Data Collection

**FIGURE 1:**
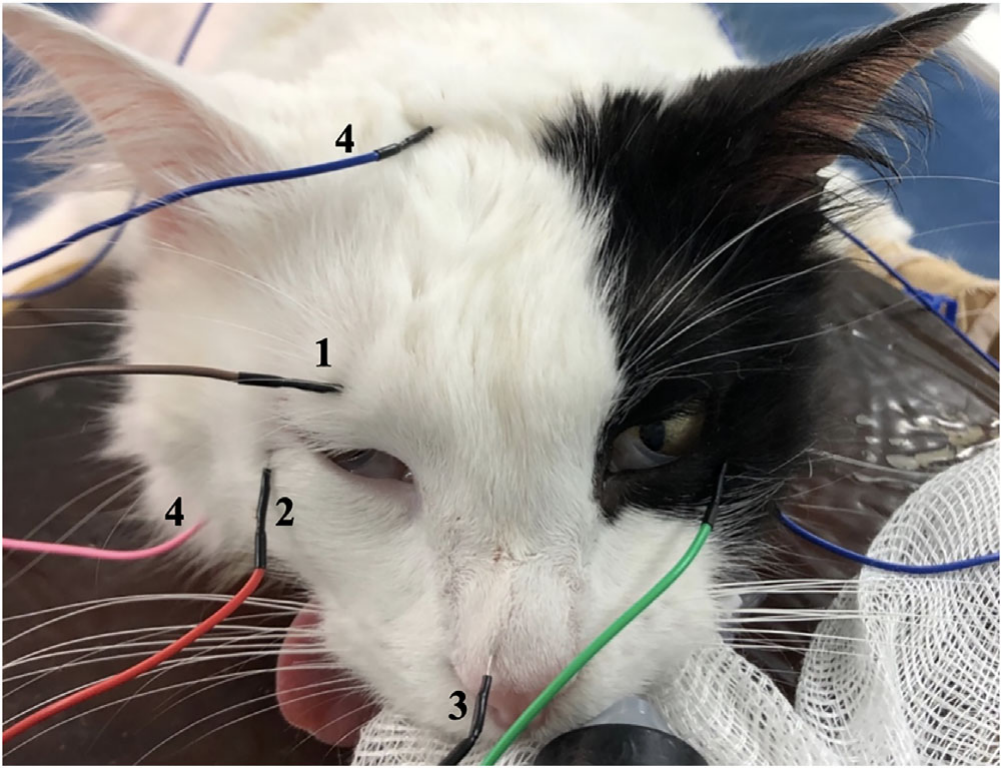
Illustration of the placement of EMG electrodes. Active electrode cathode used for supraorbital nerve stimulation (1), ipsilateral needle electrode recording from the orbicularis oculi (2), ground electrode (3), and reference electrodes (4).

The blink reflex test was performed by stimulation of the supraorbital nerve with silver needle electrodes (2–2.5 mm). The cathode was placed 1 cm dorsal to the medial canthus of the eye along the supraorbital aspect of the frontal bone where the supraorbital nerve exits the orbital cavity, and the anode was used as a reference electrode. Needle electrodes were placed on the lateral part of the right ventral eyelid to obtain recordings from the orbicularis oculi muscle. The needle electrode placed on the nose was used as ground (Figure 1).

After the vaporiser was switched off at the end of the operation, supramaximal stimuli were given in the form of 0.1 ms^2^ waves at each decreasing MAC value of 0.1 starting from 2.9 end‐tidal MAC at which the patient was in deep anaesthesia. Each stimulus was applied three times depending on the changing MAC value and the stimuli were given at certain intervals to prevent habituation of the response. The stimulus was given only from the right supraorbital nerve in each patient and recordings were obtained from the right orbicularis oculi. Latency, amplitude and duration values were recorded.

### 2.7 Statistical Analysis

Descriptive statistics related to the data were shown as “Arithmetic Mean + Std. error” for continuous variables and “*n*, %n” for categorical variables. The relationship between the variables obtained was analysed by Pearson's correlation analysis and paired *t*‐test for normally distributed data groups and Spearman's correlation analysis for non‐normally distributed data groups. In all statistical analyses, values with *p *< 0.05 were considered statistically significant. SPSS 14.01 package program was used for statistical analyses.

## 3 Results

**FIGURE 2:**
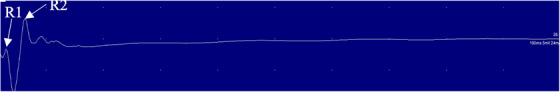
Blink reflex recording with R1 and R2 traces obtained at 0.5 minimum alveolar concentration.

All of the cases were mixed‐breed, 7 were female (21.88%) and 25 were male (78.12%). Body weights of the cases ranged between 1.8 and 5 kg (mean 3.80 ± 0.8) and ages ranged between 6 and 48 years (months) (mean 18.68 ± 11.82). R1 and R2 parameters were obtained from the patients. R1 wave showed a monophasic waveform. R2 wave showed a polyphasic waveform (Figure 2).

A total of 839 R1 latencies were obtained from all cases. The arithmetic mean of R1 of all cases was found to be 1.37. A total of 840 R2 latencies were obtained from all cases. The mean R2 latencies obtained were found to be 3.78 (Table 1). 838 R1 amplitudes were obtained from all subjects. The mean of R1 amplitudes was found to be 2705.25. 839 R2 amplitudes were obtained from all subjects. The mean R2 amplitudes were found to be 7427.01 (Table 2). Latency times of R1 and R2 were measured. In 32 cases, R1 latency durations were 841. The arithmetic mean was found to be 2.39. R2 latency durations were 842. The arithmetic mean was found to be 5.13 (Table 3). In the study, R1 and R2 parameters were obtained in all MAC values in 32 cats. The mean MAC value at which R1 and R2 were obtained last, when the animal could not tolerate electrical stimulation and woke up, was found to be 0.43 (Table 4).

**TABLE 1:** Average latency values obtained.

Variable	*n*	Arit. Mean	Std. Error
Latency R1	839	1.37	0.38
Latency R2	840	3.78	0.70

Abbreviations: Arit. Mean, arithmetic mean; *n*, number of cases; Std. Error, standard error.

**TABLE 2:** Mean amplitude values.

**Variable**	***n***	**Arit. Mean**	**Std. Error**
Amplitudes R1	838	2705.25	1743.61
Amplitudes R2	839	7427.01	4373.47

Abbreviations: Arit. Mean, arithmetic mean; *n*, number of cases; Std. Error, standard error.

**TABLE 3:** Average durations.

Variable	*n*	Arit. Mean	Std. Error
Latency D R1	841	2.39	0.71
Latency D R2	842	5.13	1.71

Abbreviations: Arit. Mean, arithmetic mean; *n*, number of cases; Std. Error, standard error.

**TABLE 4:** Average MAC value when the parameters R1 and R2 were last obtained and the cats woke up.

**Variable**	***n***	**Arit. Mean**	**Std. Error**
Average MAC value	32	0.43	0.20

Abbreviations: Arit. Mean, arithmetic mean; *n*, number of cases; Std. Error, standard error.

There was a strong positive correlation between R1 latency (*p* = 0.001) and MAC and a moderate positive correlation between R2 latency (*p* = 0.086) and MAC. There was a very strong negative correlation between R1 amplitude (*p* = 0.001) and MAC and a moderate positive correlation between R2 amplitude (*p* = 0.030) and MAC. There was a moderate negative correlation between R1 duration (*p* = 0.079) and MAC and a weak negative correlation between R2 duration (*p* = 0.244) and MAC (Table 5).

**TABLE 5:** Correlations between MAC and variables.

Latency	Variables	MAC
Latency R1	*r*	0.842
	*p*	0.001
	*n*	27
Latency R2	*r*	−0.337
	*p*	0.086
	*n*	27
Amplitude R1	*r*	−0.805
	*p*	0.001
	*n*	27
Amplitude R2	*r*	0.418
	*p*	0.030
	*n*	27
Duration R1	*r*	−0.344
	*p*	0.079
	*n*	27
Duration R2	*r*	−0.232
	*p*	0.244
	*n*	27

*Note: p* < 0.05 is statistically significant; *n*: categorical variable; *r*: correlation direction.

When the relationship between anaesthesia stages and MAC was compared, R1 amplitude and R2 amplitude were found to be significant only between stage II and awake stage. In all other stages, R1 latency, R2 latency, R1 amplitude and R2 amplitude were not significant (Table 6).

**TABLE 6:** Relationship between anaesthesia stages and latency and amplitude (R1, R2).

Groups compared	Variables	R1 latency
Deep anaesthesia medium plan II—Deep anaesthesia light plan I	*p*	0.070
Deep anaesthesia light plan I—Phase II	*p*	0.365
Phase II—Awake	*p*	0.761
		**R2 latency**
Deep anaesthesia medium plan II—Deep anaesthesia light plan I	*p*	0.555
Deep anaesthesia light plan I—Phase II	*p*	0.460
Phase II—Awake	*p*	0.157
		**R1 amplitude**
Deep anaesthesia medium plan II—Deep anaesthesia light plan I	*p*	0.070
Deep anaesthesia light plan I—Phase II	*p*	0.073
Phase II—Awake	*p*	0.015
		**R2 amplitude**
Deep anaesthesia medium plan II—Deep anaesthesia light plan I	*p*	0.101
Deep anaesthesia light plan I—Phase II	*p*	0.141
Phase II—Awake	*p*	0.037

## 4 Discussion

There appear to be significant quantitative differences between human and cat blinks in response to supraorbital nerve stimulation. At stimulus thresholds for unilateral blinks, R2 occurs unilaterally in cats (Hiraoka and Shimamura 1977; Tokunaga et al. 1958; Tamai et al. 1982) and bilaterally in humans (LeDoux et al. 1997; Kugelberge 1952; Shahani 1970). In the present study, blink responses were obtained only on the stimulus side in accordance with other cat studies.

The R1 and R2 responses have latency intervals of 9–12 and 15–25 ms (Lindquist and Mårtensson 1970) when the skin over the orbicularis oculi muscle is touched, respectively, and 7.5 + 0.5 and 15.1 + 1.4 ms (Hiraoka and Shimamura 1977) for electrical stimulation of the cornea in cats. In the authors' experiments, the latency intervals of two consecutive responses to electrical stimulation of the infraorbital nerve were 5.8–8.0 and 12.4–17.0 ms, respectively (Tamai et al. 1982). Neurophysiological studies of the blink reflex to supraorbital nerve stimulation were performed in eight awake, adult male cats. Mean (±SE) minimal latencies for R1 and R2 were 8.26 ± 0.85 and 22.97 ± 1.53 ms, respectively (LeDoux et al. 1997). Stimulus location, duration and magnitude can affect the latency and duration of R2 and, to a lesser degree, R1 (Berardelli et al. 1985). In the present study, R1 latency was 1.37 ± 0.38 and R2 latency was 3.78 ± 0.70 by stimulating the supraorbital nerve. It was thought that the latency might be affected by the position, duration and magnitude of the stimulus.

Animal and human studies have shown that an electrically evoked blink reflex is suppressed during sedation and anaesthesia (Mourısse et al. 2003, 2004). Therefore, measuring the blink reflex may reflect the depression of reflex arcs induced by anaesthetics (Mourısse et al. 2004).

The blink parameters obtained in the study were not affected by the depth of anaesthesia, and R1 and R2 blink parameters were obtained in all MAC ranges up to 0.43 ± 0.20 MAC value at which the cats woke up on average. A very strong positive correlation was obtained between R1 latency and MAC, and a moderate negative correlation was obtained between R2 latency and MAC. A very strong negative correlation was obtained between R1 amplitude and MAC, and a moderate positive correlation was obtained between R2 amplitude and MAC. A moderate negative correlation was obtained between R1 duration and MAC, and a weak negative correlation was obtained between R2 duration and MAC.

In the present study, when the relationship between the stages of anaesthesia scaled according to the Guedel classification and MAC was compared, R1 amplitude and R2 amplitude were found to be significant only between stage II and awake stage. In all other stages, R1 latency, R2 latency, R1 amplitude and R2 amplitude were not significant. It was concluded that the depth of anaesthesia can be determined only by looking at the R1 and R2 amplitudes between stage II and awake stage.

## 5 Conclusion

In this study, mean latency, amplitude and duration values in cats under sevoflurane anaesthesia were determined. It was demonstrated that it was possible to elicit blink parameters in cats under sevoflurane anaesthesia with a short stimulus sequence to the supraorbital nerve. Only two blink responses, R1 and R2, were elicited at all MAC values. The study was carried out in mongrel breeds and it was thought that species‐specific studies could be performed. The study was performed by stimulating the supraorbital nerve and it was thought that other nerves could be stimulated and compared.

## Author Contributions

**Özlem Şengöz Şirin**: conceptualisation, investigation, funding acquisition, writing – original draft. **Ayşe Nihan Elvan**: formal analysis.

## Conflicts of Interest

The authors declare no conflicts of interest.

### Peer Review

The peer review history for this article is available at https://www.webofscience.com/api/gateway/wos/peer‐review/10.1002/vms3.70366.

## Data Availability

The data that support the findings of this study are available from the corresponding author upon reasonable request.
